# Supplementary material for: Rare t(X;14)(q28;q32) translocation reveals link between MTCP1 and chronic lymphocytic leukemia
Source: Nat Commun. 2021 Nov 3;12:6338. doi: 10.1038/s41467-021-26400-x (PMC8566464; doi:10.1038/s41467-021-26400-x)
Supplement: Supplementary file 1 — Supplementary Information [file 41467_2021_26400_MOESM1_ESM.pdf]

Supplementary Figures & Figure Legends

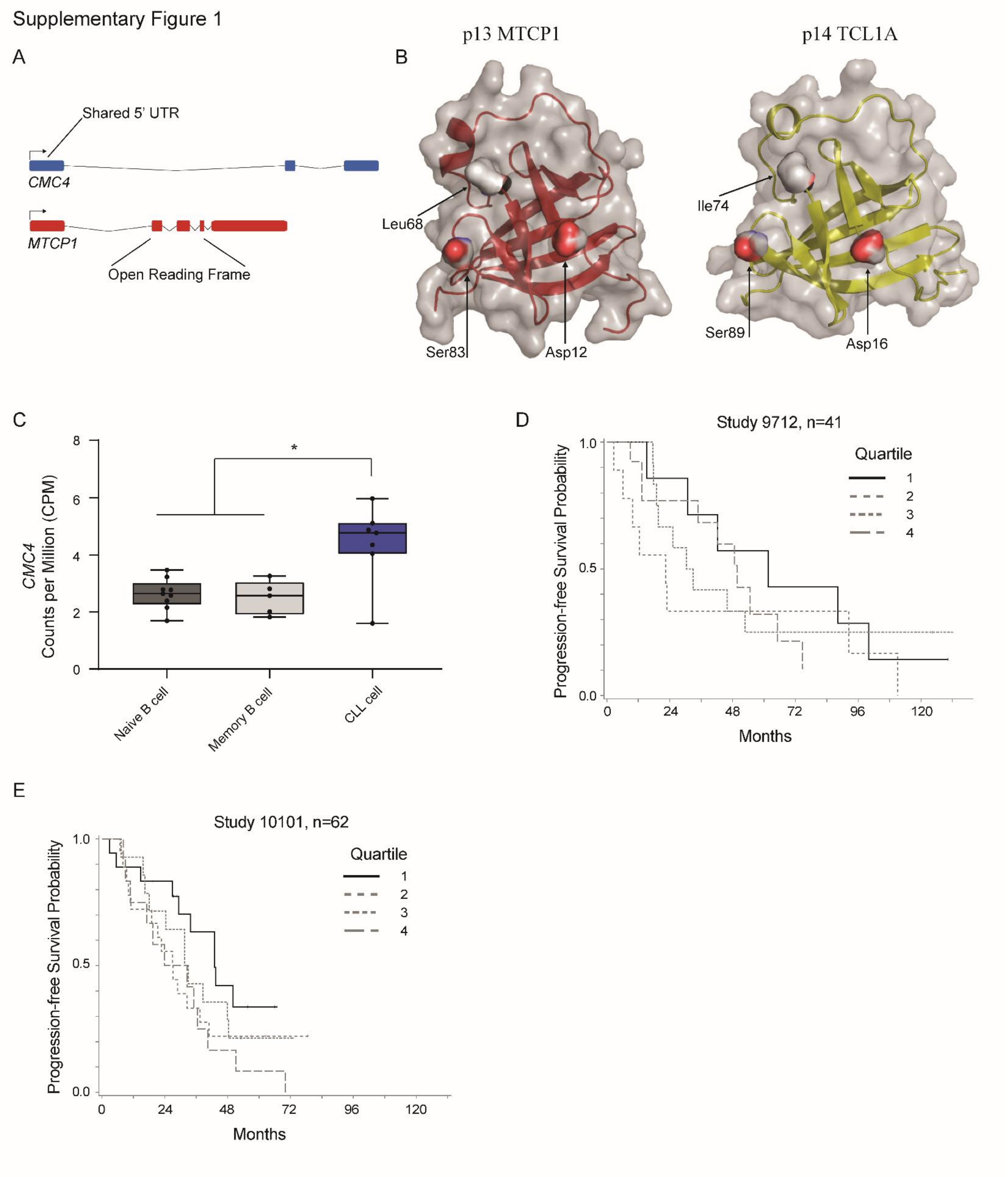

Supplementary Figure 1. *MTCP1*, splice variant *CMC4*, and p13 MTCP1 homolog TCL1A

(a) Genomic arrangement of the *MTCP1* locus on the reverse strand of the X chromosome. Bicistronism from an ancient insertion event yields two isoforms. The truncated isoform is termed "*CMC4.*" The longer *MTCP1* isoform is a member of the *TCL1* proto-oncogene family and is amplified in t(X;14)-associated T cell leukemia.

(b) 3-Dimensional surface rendering with beta-barrel tertiary structures as determined via crystal structure analysis showing high degree of overlap between human p13 MTCP1 and p14 TCL1A (RCSB IDs: 1A1X, 1JSG). Surface residues Asp16, Ile74, and Ser89 have shown to be critical for TCL1A-AKT interactions, and biochemical conservation of these residues on the surface of p13 MTCP1 (Asp12, Leu68, Ser83) suggests shared AKT-binding abilities between family members. Space fill rending of these conserved amino acid residues possibly necessary for AKT interactions are highlighted.

(c) *CMC4* mRNA expression is elevated in CLL cells compared to naïve- or memory-B cell subsets (p=0.0140, p=0.0105, respectively). Data obtained from the Blueprint database. Box elements reflect 2nd - 3rd quartile, center line reflects the median value, and whiskers reflect the distance from the upper and lower limit to the box elements. Naïve B cells – dark grey/left, n=9; memory B cells – light grey/middle, n=5; CLL cells – blue/right, n=7. p-value estimated using a two-tailed unpaired t-test with Welch's correction.

(d) Progression-free survival (PFS) by quartile of *MTCP1* expression is visualized via Kaplan-Meier plot for a subset of CLL patients from a Cancer and Leukemia Group B clinical trial (9712; n=41).

(e) Progression-free survival by quartile of *MTCP1* expression is visualized via Kaplan-Meier plot for a subset of CLL patients from a Cancer and Leukemia Group B clinical trial (10101; n=62).

Supplementary Figure 2

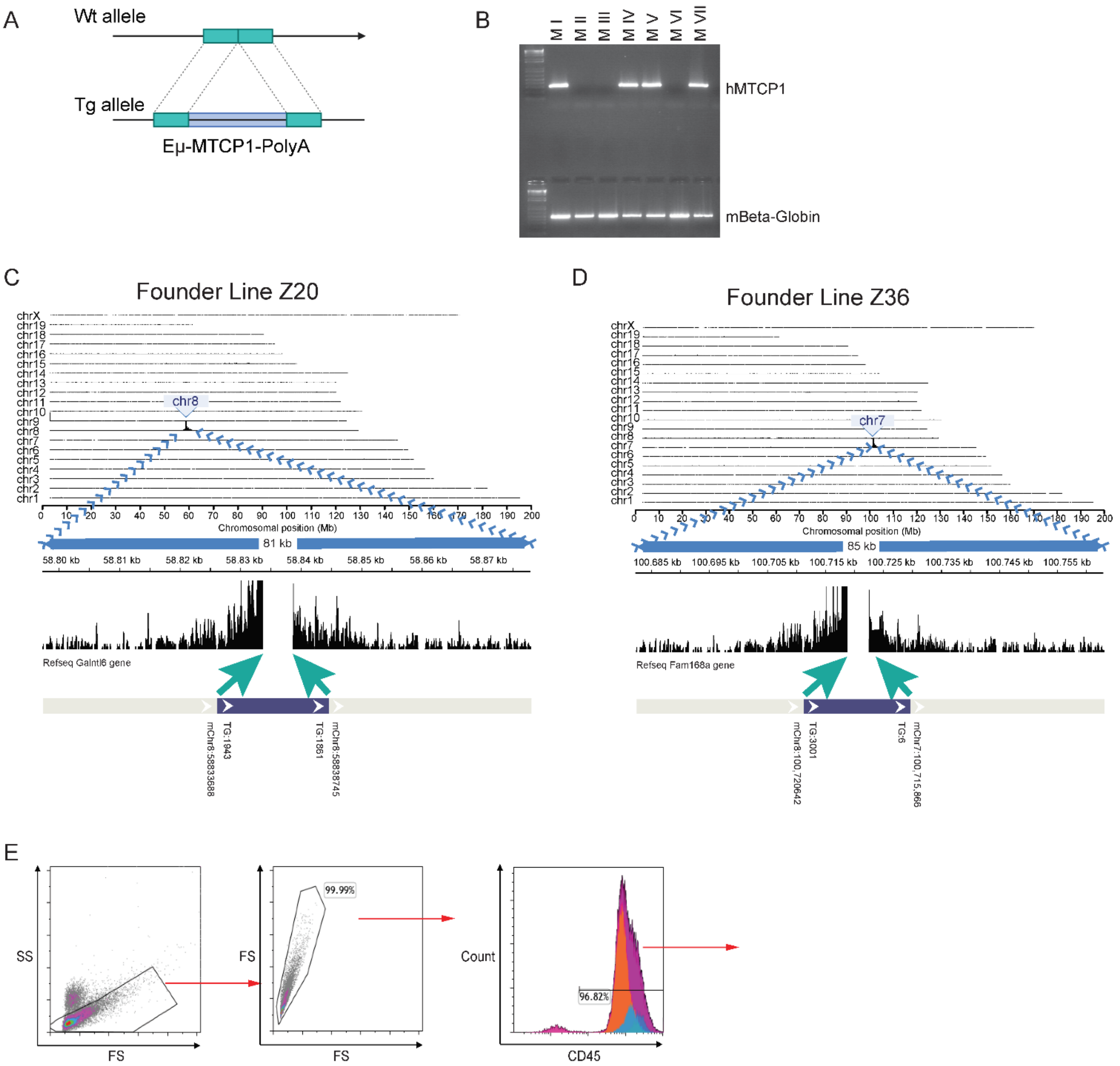

Supplementary Figure 2. Generating the Eμ-MTCP1 mouse

(a) Diagram illustrating the transgenic allele of the Eμ-MTCP1 mouse. Human recombinant MTCP1 is constitutively expressed under control of VH promoter-IgH-Eμ enhancer elements for expression restricted to the B cell compartment. Using this transgenic engineering model, five independent founder lines were generated. Artistic rendering created with Biorender.com

(b) Successful passage of the hMTCP1 transgene from Eμ-MTCP1 founder mice is observed in progeny. PCR analysis was used to confirm the integration of the hMTCP1 transgene into the genome of subsequent generations of Eμ-MTCP1 littermate mice (M I-VII). A band of 340bp indicates the presence of the transgenic MTCP1 allele. Mouse beta-globin was used as amplification control sequence, generating a band of ~495bp. Images represent three independent tests.

(c) Mapping analysis of the hMTCP1 transgene integration site in Eμ-MTCP1 mice derived from the Z20 founder was performed via targeted locus amplification. Next generation sequencing was performed and reads were aligned to mouse genome version GRCm10. The hMTCP1 transgene integrated at chr8:58,833,688-58,838,745 in an intron of the Galntl6 gene. >10 copies of the transgene have integrated in this locus.

(d) Mapping analysis of the hMTCP1 transgene integration site in Eμ-MTCP1 mice derived from the Z36 founder was performed via targeted locus amplification. Next generation sequencing was performed and reads were aligned to mouse genome version GRCm10. The hMTCP1 transgene integrated at chr7:100,715,866-100,720,642 within an intron of the Fam168a gene. >10 copies of the transgene have integrated in this locus.

(e) Representative gating strategy used to identify B lymphocytes from single Cd45+ cell populations circulating in the peripheral blood of Eμ-MTCP1 mice.

Supplementary Figure 3

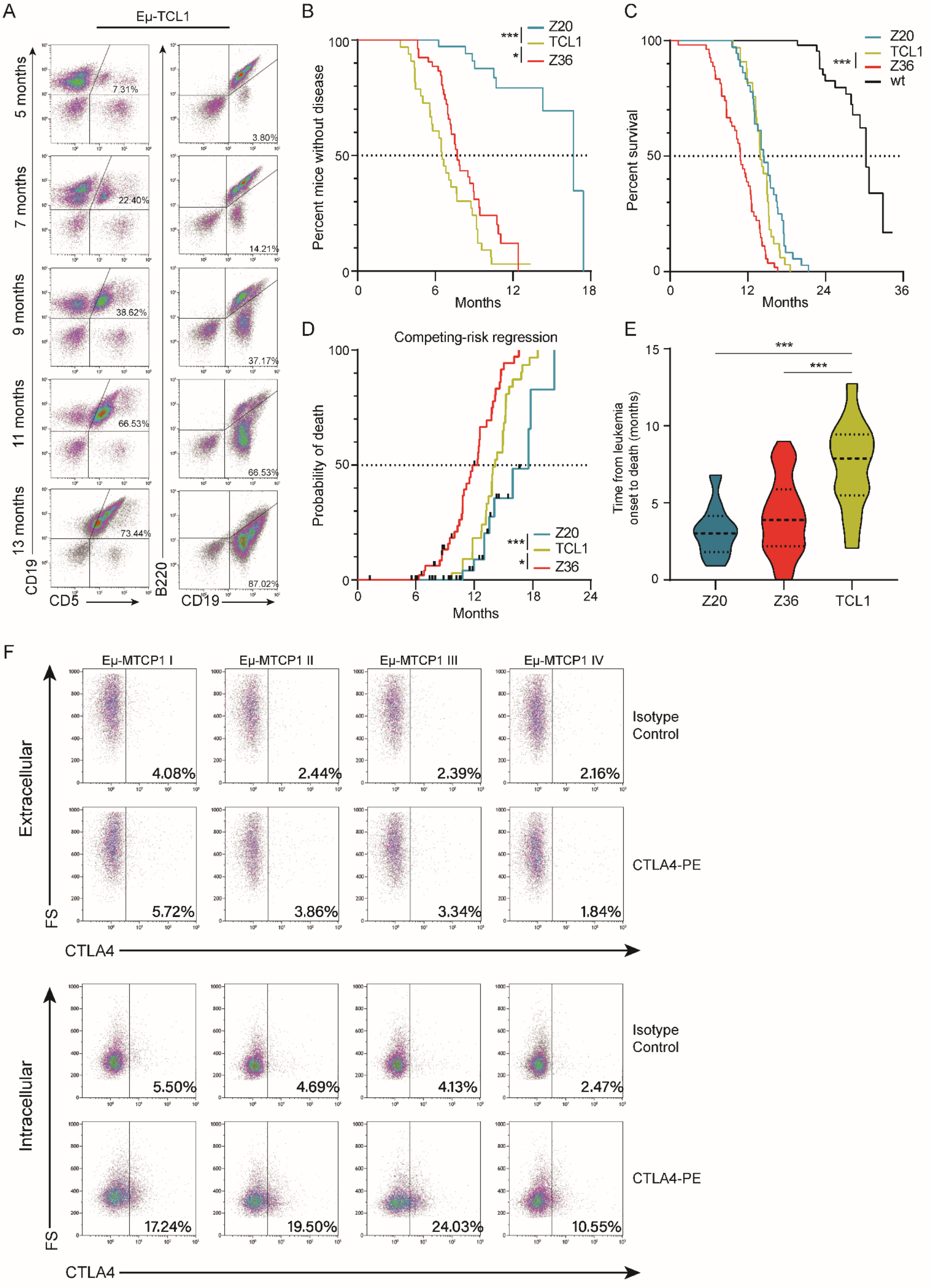

Supplementary Figure 3. Eμ-MTCP1 mice develop a leukemia resembling murine and human CLL

(a) Longitudinal flow cytometry analysis of Eμ-TCL1 mice showing progressive development of a $CD5^+/CD19^+$ and $CD19^+$/B220dim $CD45^+$ CLL-like population in the blood.

(b) Kaplan-Meier estimation of median time to disease onset in Eμ-MTCP1 (Z36, n=54; Z20, n=36) and Eμ-TCL1 mice (n=33). Median time from birth to disease onset was shorter in Eμ-TCL1 mice (6.5 months) than Eμ-MTCP1 mice (p=0.023 and p<0.001 for Z36 (7.7 months) and Z20 (16.7 months), respectively). Disease onset was defined as >20% expansion of $CD5^+/CD19^+$ and $CD19^+$/B220dim $CD45^+$ cell populations in the blood determined by flow cytometry.

(c) Kaplan-Meier estimation of median survival in Eμ-MTCP1 (Z36, n=54; Z20, n=36) vs Eu-TCL1 mice (n=33). The median survival time was 10.8 months (95% CI: 9.5-12.5) for Eμ-MTCP1 founder line Z36 and 14.5 months (95% CI: 13.2-16) for founder line Z20, compared to 13.9 months (95% CI: 13.3-15) for Eμ-TCL1 mice (log-rank p<0.001 and p=0.113 for Z36 and Z20, respectively). Representative survival of wildtype mice (n=54) is shown as reference. P-values (in **b** and **c**) determined by estimates from a Cox proportional hazards model.

(d) Competing-risk assessment to estimate the median survival in Eμ-MTCP1 (Z36 and Z20) and Eμ-TCL1 mice. Mice were censored (black mark on curve) at the time at which a T cell or myeloid cell abnormality were observed. The median estimated survival time was 12.4 months for Eμ-MTCP1 found Z36 (n=54) and 17.6 months for Eμ-MTCP1 founder Z20 (n=36). The Median estimated survival for Eμ-TCL1 mice was 14.1 months (n=33). "***" represents p<0.001 and "*" represents p=0.0503 using a two-tailed unpaired t-test with Welch's correction.

(e) Evaluation of time between CLL onset (defined in **b**) and time of death in Eμ-MTCP1 (Z36, n=38; Z20, n=10) and Eμ-TCL1 (n=32) mice. Reduced time from disease onset to death reflects the delayed onset but rapid disease course in Eμ-MTCP1 mice. "***" represents p<0.001 using a two-tailed unpaired t-test with Welch's correction.

(f) Intracellular CTLA-4 expression in CLL-like cells from Eμ-MTCP1 mice (founder Z36) resembles that of human CLL. CTLA-4 staining and analysis of peripheral blood cells via flow cytometry reveals elevated intracellular expression in Eμ-MTCP1 mice while lacking surface expression (n=4).

Supplementary Figure 4

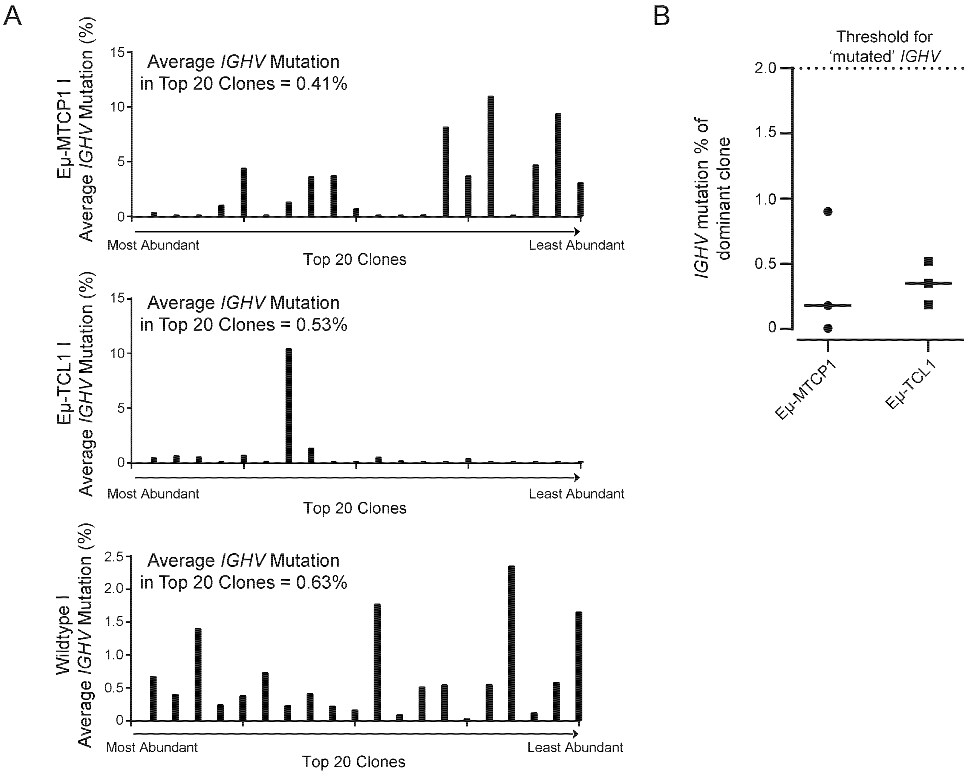

Supplementary Figure 4. Low IGHV mutation burden observed in Eμ-MTCP1 and Eμ-TCL1 B lymphocytes

(a) Mutation status of the 20 most abundant IGHV genes in Eμ-MTCP1, Eμ-TCL1, and non-transgenic mice (data shown from one mouse is representative of n=3 per group). The vast majority of IGHV reads in the dominant clones from each mouse revealed a low mutational burden. IGHV gene sequences determined from RNA-sequencing were compared against germline controls to assess mutation frequency. The X-axis represents the analyzed clone from most to least abundant. Y-axis represents mutation frequency per clone.

(b) IGHV gene sequences determined from RNA-sequencing were compared against germline controls to assess mutation frequency in Eμ-MTCP1 and Eμ-TCL1 spleen cells. A low mutation rate in the *IGHV* region was identified in Eμ-MTCP1 and Eμ-TCL1 cells, well below the threshold (>2%) for classification of "mutated *IGHV*."

Supplementary Figure 5

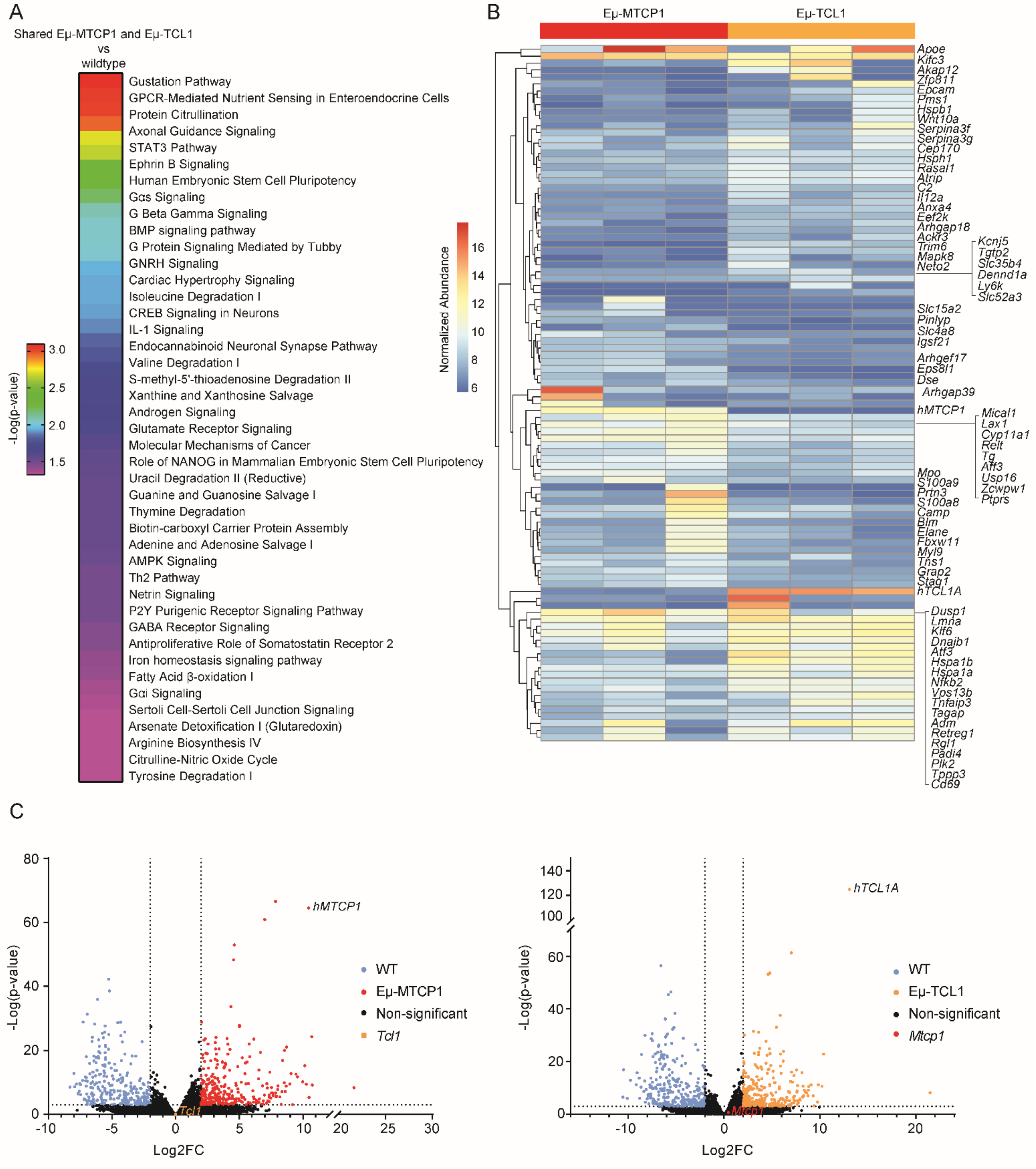

Supplementary Figure 5. Transcriptional profile in Eμ-MTCP1 and Eμ-TCL1 mice

(a) Ingenuity pathway analysis (IPA) of significantly enriched genes (Log2FC>2, p-value<0.001) shared between Eμ-MTCP1 and Eμ-TCL1 transgenic strains when compared to wildtype littermates. Rainbow color scale reflects –Log(p-value) determined via Fisher's exact test. Complete list of IPA terms and p-values are available in the supplemental file: source data.

(b) The top 100 most-variable genes between Eμ-MTCP1 and Eμ-TCL1 mice (n=3 per group) visualized via heatmap. IGH genes are removed for clarity. Genes listed on the y-axis are arranged via hierarchical clustering using Euclidean distance measurements. Heat map scale is representative of normalized Log2 expression between all samples.

(c) Differential mRNA expression analysis of splenic B cells from Eμ-MTCP1, Eμ-TCL1, and wildtype mice visualized via volcano plot (n=3 per group). 275 genes enriched in wildtype mice (blue) and 301 genes enriched in Eμ-MTCP1 mice (red) are shown (left panel). 335 genes enriched in wildtype mice (blue) and 445 genes enriched in Eμ-TCL1 mice (yellow) are shown (right panel; Log2FC >2; p-value <0.001). Differential expression of the *Mtcp1 and Tcl1* genes are highlighted. P-value determined in DESeq2 via Wald test. Complete list of genes and p-values are available in the supplemental file: source data.

Supplementary Tables

Supplementary Table 1: Stratified proportional hazards models[A] for progression-free survival

Variable	Stratified Models for Each Variable	Stratified Model including All Variables with $p \leq 0.20$[B]
	p (HR, 95% CI)	p (HR, 95% CI)
MTCP1 Expression, 2-fold increase	0.03 (2.09, 1.08-4.05)	0.32 (1.40, 0.72-2.69)
Zap-70 Methylation, <20 vs. ≥20	0.05 (1.87, 1.01-3.46)	0.31 (1.40, 0.73-2.70)
IgHV, Unmutated vs Mutated	0.67 (1.12, 0.66-1.91)	---
Cytogenetics, del(17p)/del(11q) vs. Other	0.01 (2.11, 1.20-3.72)	0.003 (2.46, 1.37-4.39)
Age, 10-year increase	0.27 (0.88, 0.71-1.10)	---
Gender, Female vs. Male	0.12 (0.64, 0.36-1.12)	0.12 (0.63, 0.35-1.13)
Hemoglobin, 1 unit increase	0.85 (0.99, 0.89-1.10)	---
WBC, 2-fold increase	0.01 (1.31, 1.06-1.62)	0.02 (1.31, 1.05-1.63)
Rai Stage (III/IV vs. I/II)	0.89 (0.97, 0.61-1.53)	---
Performance Status (1/2 vs. 0)	0.43 (1.20, 0.76-1.89)	---

[A] Stratified on study cohort.
[B] Each predictor is adjusted for all others in the model.
HR = hazard ratio, CI = confidence interval
P-values are from two-sided t-tests, obtained from stratified Cox proportional hazards models combined across 20 imputed datasets. P-values were not adjusted for multiple testing.

Supplementary Table 2: Stratified proportional hazards models* for progression-free survival

Variable	Stratified Models for Each Variable	Stratified Model including *MTCP1* Expression and *IgHV*[+]
	p (HR, 95% CI)	p (HR, 95% CI)
MTCP1 Expression, 2-fold increase	0.03 (2.09, 1.08-4.05)	0.03 (2.08, 1.07-4.05)
IgHV, Unmutated vs Mutated	0.67 (1.12, 0.66-1.91)	0.87 (1.05, 0.61-1.80)

*Stratified on study cohort.
[+]Each predictor is adjusted for all others in the model.
HR = hazard ratio, CI = confidence interval
P-values are from two-sided t-tests, obtained from stratified Cox proportional hazards models combined across 20 imputed datasets. P-values were not adjusted for multiple testing.

Supplementary Table 3: IG V gene usage in Eμ-MTCP1 mice

IgHv Gene Usage

**Eμ-MTCP1 I**		**Eμ-MTCP1 II**		**Eμ-MTCP1 III**		**Eμ-TCL1 I**		**Eμ-TCL1 II**		**Eμ-TCL1 III**		**Wildtype I**		**Wildtype II**		**Wildtype III**	
IGHV1-55	66.12%	IGHV12-3	94.92%	IGHV5-4	72.69%	IGHV1-50	70.82%	IGHV1-15	80.96%	IGHV12-3	97.16%	IGHV5-4	7.06%	IGHV5-17	6.02%	IGHV5-17	14.98%
IGHV1-50	13.83%	IGHV2-3	1.10%	IGHV5-12	13.41%	IGHV1-55	6.16%	IGHV12-3	10.46%	IGHV11-2	0.92%	IGHV6-3	3.99%	IGHV4-1	5.22%	IGHV1-53	5.96%
IGHV1-61	4.19%	IGHV1-82	0.49%	IGHV14-1	6.56%	IGHV1-61	3.73%	IGHV1-7	2.73%	IGHV5-12	0.54%	IGHV1-53	3.81%	IGHV1-53	3.96%	IGHV3-6	4.31%
IGHV1-62-3	2.89%	IGHV7-3	0.47%	IGHV5-15	2.08%	IGHV1-19	3.24%	IGHV11-2	0.65%	IGHV6-3	0.33%	IGHV3-6	3.79%	IGHV1-80	3.66%	IGHV6-3	2.82%
IGHV1-19	2.26%	IGHV1-78	0.41%	IGHV5-17	1.09%	IGHV1-36	3.22%	IGHV1-5	0.59%	IGHV5-17	0.11%	IGHV4-1	3.17%	IGHV6-3	3.57%	IGHV1-18	2.65%
IGHV1-36	2.02%	IGHV5-12	0.36%	IGHV7-1	0.68%	IGHV14-2	2.53%	IGHV1-55	0.49%	IGHV11-1	0.08%	IGHV1-80	2.74%	IGHV1-26	3.28%	IGHV5-4	2.57%
IGHV1-18	1.44%	IGHV11-2	0.20%	IGHV5-12-4	0.58%	IGHV1-62-3	2.15%	IGHV1-23	0.28%	IGHV1-26	0.08%	IGHV1-72	2.59%	IGHV9-3	2.92%	IGHV1-75	2.49%
IGHV1-59	1.40%	IGHV6-3	0.13%	IGHV5-16	0.48%	IGHV1-59	1.30%	IGHV1-50	0.25%	IGHV5-15	0.08%	IGHV8-5	2.56%	IGHV5-9-1	2.81%	IGHV9-3	2.47%
IGHV1-52	1.10%	IGHV4-1	0.13%	IGHV1-62-2	0.39%	IGHV1-18	1.28%	IGHV1-19	0.21%	IGHV5-5	0.06%	IGHV5-17	2.54%	IGHV8-12	2.68%	IGHV1-55	2.43%
IGHV5-4	0.50%	IGHV5-15	0.12%	IGHV5-6	0.37%	IGHV1-62-2	1.26%	IGHV1-18	0.18%	IGHV1-50	0.05%	IGHV5-16	2.50%	IGHV2-9-1	2.47%	IGHV1-26	2.23%
Others	*4.24%*	*Others*	*1.67%*	*Others*	*1.68%*	*Others*	*4.31%*	*Others*	*3.19%*	*Others*	*0.59%*	*Others*	*65.26%*	*Others*	*63.41%*	*Others*	*57.09%*

IgKv Gene Usage

**Eμ-MTCP1 I**		**Eμ-MTCP1 II**		**Eμ-MTCP1 III**		**Eμ-TCL1 I**		**Eμ-TCL1 II**		**Eμ-TCL1 III**		**Wildtype I**		**Wildtype II**		**Wildtype III**	
IGKV12-89	97.19%	IGKV4-91	97.79%	IGKV4-91	79.86%	IGKV10-94	93.47%	IGKV8-30	65.60%	IGKV4-91	93.21%	IGKV1-117	9.16%	IGKV6-15	6.48%	IGKV3-4	12.90%
IGKV4-91	0.44%	IGKV8-19	0.44%	IGKV19-93	17.92%	IGKV8-30	4.22%	IGKV4-91	19.38%	IGKV5-43	4.04%	IGKV6-15	4.61%	IGKV1-117	5.36%	IGKV10-96	4.82%
IGKV12-44	0.26%	IGKV6-20	0.24%	IGKV5-39	0.51%	IGKV6-29	0.55%	IGKV6-29	7.16%	IGKV14-126	0.98%	IGKV1-110	3.64%	IGKV1-110	4.66%	IGKV5-39	4.72%
IGKV6-20	0.24%	IGKV14-126	0.17%	IGKV3-11	0.36%	IGKV10-95	0.36%	IGKV14-126	1.28%	IGKV6-20	0.39%	IGKV8-27	3.58%	IGKV3-4	4.35%	IGKV1-110	3.57%
IGKV6-15	0.18%	IGKV14-111	0.12%	IGKV6-25	0.10%	IGKV6-20	0.13%	IGKV4-86	0.85%	IGKV12-44	0.25%	IGKV1-135	3.52%	IGKV6-23	4.12%	IGKV6-15	3.56%
IGKV8-27	0.16%	IGKV1-117	0.11%	IGKV2-109	0.09%	IGKV3-11	0.11%	IGKV3-11	0.47%	IGKV6-15	0.23%	IGKV6-23	3.29%	IGKV6-20	3.83%	IGKV1-117	3.47%
IGKV17-121	0.10%	IGKV1-110	0.09%	IGKV12-44	0.08%	IGKV12-89	0.09%	IGKV4-81	0.39%	IGKV12-89	0.11%	IGKV10-96	2.90%	IGKV8-27	3.77%	IGKV4-59	2.85%
IGKV3-11	0.08%	IGKV5-39	0.07%	IGKV1-110	0.07%	IGKV14-126	0.08%	IGKV12-89	0.30%	IGKV3-11	0.09%	IGKV8-30	2.69%	IGKV5-39	3.76%	IGKV14-126	2.65%
IGKV4-59	0.07%	IGKV12-89	0.07%	IGKV6-20	0.06%	IGKV6-15	0.05%	IGKV5-39	0.28%	IGKV10-94	0.07%	IGKV4-59	2.60%	IGKV1-135	3.20%	IGKV6-23	2.62%
IGKV14-126	0.06%	IGKV6-15	0.05%	IGKV14-126	0.05%	IGKV4-91	0.04%	IGKV8-19	0.24%	IGKV4-77	0.05%	IGKV8-24	2.33%	IGKV10-96	3.18%	IGKV4-57	2.46%
Others	*1.21%*	*Others*	*0.85%*	*Others*	*0.89%*	*Others*	*0.91%*	*Others*	*4.06%*	*Others*	*0.58%*	*Others*	*61.68%*	*Others*	*57.29%*	*Others*	*56.37%*

IgLv Gene Usage

**Eμ-MTCP1 I**		**Eμ-MTCP1 II**		**Eμ-MTCP1 III**		**Eμ-TCL1 I**		**Eμ-TCL1 II**		**Eμ-TCL1 III**		**Wildtype I**		**Wildtype II**		**Wildtype III**	
IGLV1	0.83997	IGLV1	0.93315	IGLV1	0.90244	IGLV1	0.91767	IGLV1	0.98534	IGLV1	0.79764	IGLV1	0.6172	IGLV1	0.65981	IGLV1	0.63037
IGLV3	0.12596	IGLV2	0.04013	IGLV3	0.07012	IGLV3	0.06769	IGLV3	0.00831	IGLV3	0.18316	IGLV2	0.30255	IGLV2	0.21153	IGLV2	0.30545
IGLV2	0.03406	IGLV3	0.02672	IGLV2	0.02744	IGLV2	0.01464	IGLV2	0.00635	IGLV2	0.0192	IGLV3	0.08025	IGLV3	0.12866	IGLV3	0.06417
