# Supplementary material for: Rare t(X;14)(q28;q32) translocation reveals link between MTCP1 and chronic lymphocytic leukemia
Source: Nat Commun. 2021 Nov 3;12:6338. doi: 10.1038/s41467-021-26400-x (PMC8566464; doi:10.1038/s41467-021-26400-x)
Supplement: Supplementary file 3 — Reporting summary [file 41467_2021_26400_MOESM3_ESM.pdf]

nature portfolio

Corresponding author(s): Dr. Rosa Lapalombella

Last updated by author(s): Sep 1, 2021

Reporting Summary

Nature Portfolio wishes to improve the reproducibility of the work that we publish. This form provides structure for consistency and transparency in reporting. For further information on Nature Portfolio policies, see our Editorial Policies and the Editorial Policy Checklist.

Statistics

For all statistical analyses, confirm that the following items are present in the figure legend, table legend, main text, or Methods section.

n/a	Confirmed	
☐	☒	The exact sample size (*n*) for each experimental group/condition, given as a discrete number and unit of measurement
☐	☒	A statement on whether measurements were taken from distinct samples or whether the same sample was measured repeatedly
☐	☒	The statistical test(s) used AND whether they are one- or two-sided<br>*Only common tests should be described solely by name; describe more complex techniques in the Methods section.*
☐	☒	A description of all covariates tested
☐	☒	A description of any assumptions or corrections, such as tests of normality and adjustment for multiple comparisons
☐	☒	A full description of the statistical parameters including central tendency (e.g. means) or other basic estimates (e.g. regression coefficient) AND variation (e.g. standard deviation) or associated estimates of uncertainty (e.g. confidence intervals)
☐	☒	For null hypothesis testing, the test statistic (e.g. *F*, *t*, *r*) with confidence intervals, effect sizes, degrees of freedom and *P* value noted<br>*Give P values as exact values whenever suitable.*
☒	☐	For Bayesian analysis, information on the choice of priors and Markov chain Monte Carlo settings
☐	☒	For hierarchical and complex designs, identification of the appropriate level for tests and full reporting of outcomes
☒	☐	Estimates of effect sizes (e.g. Cohen's *d*, Pearson's *r*), indicating how they were calculated

Our web collection on statistics for biologists contains articles on many of the points above.

Software and code

Policy information about availability of computer code

Data collection

1744 suspected CLL specimens collected between November 2003 and December 2014 at The Ohio State University Comprehensive Cancer Center were evaluated for possible Xq28 rearrangements by screening metaphase karyotypes collected at time of initial biopsy.

Fluorescent in-situ Hybridization (FISH) was done according to the manufacturer's recommendations, except prior to hybridization slides were pretreated with pepsin and postfix solution. Co-denaturation of probe and sample was done on HyBrite (Abbott Molecular, Downers Grove, IL) for 5 minutes at 73 C. FISH signals were viewed using a fluorescent microscope (Zeiss axioscope 40) equipped with appropriate filters and analyzed with Applied Imaging System.

Transgenic mouse mapping and gene integration services were provided by Taconic Biosciences via a collaboration between Taconic and Cergentis. Sample preparation was carried out at Taconic Biosciences and both TLA and data analysis were carried out at Cergentis, with genotyping assay recommendations subsequently provided by Taconic.

Immunophenotyping was conducted using Beckman Coulter Gallios 3-laser-10-color (B5-R3-V2) cell analyzer. All flow cytometry data were analyzed using KALUZA v2.0 software (Becton Dickinson).

Histopathologic analysis were conducted on spleen, thymus, liver, mesenteric lymph node, and bone marrow from Eμ-MTCP1 mice meeting predefined euthanasia criteria. Tissues were fixed in 10% neutral buffered formalin (NBF). Bones were decalcified in formic acid (Surgipath). All tissues were embedded in paraffin and sectioned at 4μM onto glass slides. Photographs were taken using an Olympus SC30 camera with an Olympus BX53 microscope.

RNA-sequencing libraries were prepared with the Illumina Tru-Seq stranded kit and sequenced on a Hiseq 4000 targeting 40x10^6 fragments per sample. Transcript-level abundances were estimated using Salmon with the gencode mouse release 23, imported using tximport, with normalization and differential expression computed with DESeq2. Data processing was performed according to the CLEAR workflow, which

identifies reliably quantifiable transcripts in low-input RNA-seq for differentially expressed gene (DEG) transcripts using gene coverage profiles. MiXCR (v3.0.5) was used with default parameters except the rnaseq alignment was replaced with kaligner2 to identify preprocessed reads containing CDR3 regions from B-cell heavy, kappa, and lambda chains, generating a list of unique CDR3 sequences associated with their relative abundances and specific V(D)J gene usage. MiXCR then generates a list of unique CDR3 sequences associated with their relative abundances and specific V(D)J gene usage. To verify expression of the human MTCP1 and TCL1 transgene (hMTCP1 & hTCL1) in mice, transcript level abundances were estimated using Salmon with a modified gencode mouse reference that contained sequences from human MTCP1 and human TCL1 genes extracted from the grch38 human reference.

Artistic renderings were created and exported under a paid subscription with Biorender.com. Biologic assembly of the proposed 3-dimensional structure of MTCP1 and TCL1 proteins, as determined by x-ray diffraction protein crystallography, was visualized and exported under a University supported subscription using the PYMOL Molecular Graphics System, Version 2.3.5. Unless otherwise noted, data were visualized using GraphPad Prism version 8.3.1 for Windows, GraphPad Software, San Diego, California USA.

Data analysis

Unless otherwise noted, analyses were performed by independent statisticians within the OSU Center for Biostatistics according to methods from previously described models. All analyses were performed using SAS/STAT software, version 9.4 (SAS Institute, Inc., Cary, NC). Evaluation of the difference in mean gene expression between cell types collected from the Blueprint DCC gene expression portal generated a two-tailed p-value using an unpaired t-test with Welch's correction.
For patient data, associations between MTCP1 expression grouped by quartile and demographic, clinical, and molecular features were assessed using Fisher's exact and Kruskal-Wallis tests. MTCP1 expression was correlated with PFS using a Cox stratified proportional hazards model, stratified on study cohort. Further modeling was performed controlling for other important demographic, clinical, or molecular variables. Multiple imputation estimated missing data and combined results for 20 datasets. All p-values were two-sided and p-values less than 0.05 were considered statistically significant. No control for multiple comparisons were made. The correlation between MTCP1 expression and PFS was visualized using Kaplan-Meier plots, grouping patients into quartiles according to MTCP1 expression.
For mouse survival experiments, survival curve estimates for both overall survival (OS) and time to disease onset were calculated using the Kaplan-Meier method and differences in curves were initially assessed using the log-rank test. Next, hazard ratios (HR) and 95% CI were obtained from Cox proportional hazards models to evaluate differences between founder lines/Eu-TCL1 mice or treatment groups. Mixed effects models were used to assess changes in disease burden over time. Where applicable, data were log-transformed to reduce skewness.

For manuscripts utilizing custom algorithms or software that are central to the research but not yet described in published literature, software must be made available to editors and reviewers. We strongly encourage code deposition in a community repository (e.g. GitHub). See the Nature Portfolio guidelines for submitting code & software for further information.

Data

Policy information about availability of data

All manuscripts must include a data availability statement. This statement should provide the following information, where applicable:

- Accession codes, unique identifiers, or web links for publicly available datasets
- A description of any restrictions on data availability
- For clinical datasets or third party data, please ensure that the statement adheres to our policy

MTCP1 and CMC4 gene sequences were obtained and visualized from the Ensembl genome browser. The proposed 3-dimensional crystal structure for MTCP1 and TCL1A protein sequences were obtained from the RCSB Protein Data Bank (IDs: 1A1X [https://www.rcsb.org/structure/1A1X], 1JSG [https://www.rcsb.org/structure/1JSG], respectively). Crystal structures were determined via x-ray diffraction and presented at 2.00 Å and 2.50 Å resolution, respectively.

Gene expression data for MTCP1 and CMC4 determined from RNA-sequencing were collected from the Blueprint DCC data portal. The RNA-sequencing data generated in this study have been deposited in the GEO database under accession code #GSE176094 [https://www.ncbi.nlm.nih.gov/geo/query/acc.cgi?acc=GSE176094].

The CALGB study '10101' and '9712' data used in this are not publicly available but can be accessed from the authors of these studies. All other source data are provided as a supplementary file.

Field-specific reporting

Please select the one below that is the best fit for your research. If you are not sure, read the appropriate sections before making your selection.

[x] Life sciences   [ ] Behavioural & social sciences   [ ] Ecological, evolutionary & environmental sciences

For a reference copy of the document with all sections, see nature.com/documents/nr-reporting-summary-flat.pdf

Life sciences study design

All studies must disclose on these points even when the disclosure is negative.

Sample size

To identify potentially rare genetic lesions in CLL patients, we screened a large cohort of specimens collected on the basis of suspected CLL between November 2003 and December 2014 at The Ohio State University Comprehensive Cancer Center (total of 11 years, n=1744).

For Eμ-MTCP1 mouse colonies, prior consultation with The Ohio State University Center for Biostatistics determined a samples size of n=40 was sufficient to provide over 80% power to detect a 20% difference in overall mortality between groups, using a two-sided chi-square test with an $\alpha=0.05$ level of significance.

Histopathology images and flow cytometry plots are representative of experiments with sample size n≥3 per group. Sample size of n≥3 per

	group were deemed sufficient following assessment of the overall phenotype observed between mouse colonies. This same rationale was used to determine n=3 per group for RNA-sequencing experiments. For engraftment and therapeutic studies, similar rationale determined a sample size of n=9 was sufficient to detect response between groups.
Data exclusions	Pre-established protocols excluded from analysis any mice that perished or met removal criteria due to severe fighting wounds or malocclusion. Mice not successfully engrafted will CLL cells were excluded for analysis according to pre-established protocols.
Replication	FISH images represent three independent tests and were verified by two independent pathologists. Eμ-MTCP1 mice from two independent founder lines were followed over several generations for characterization and all replication attempts to verify experimental findings, including engraftment and therapeutic studies, amongst animal models were successful.
Randomization	CLL patients were evenly distributed into quartiles by MTCP1 gene expression for thorough assessment of the impact on survival outcomes. Organs for histopathology analysis were harvested from Eμ-MTCP1 mice meeting predefined euthanasia criteria and representative tissue samples were randomly selected from a pool of mice having evidence of a CLL-like disease. Animals enrolled in therapeutic studies were randomly assigned to treatment groups by cages. in engraftment studies, mice were randomly assigned to treatment groups according to the data which their circulating leukemia exceeded the 20% threshold and evenly distributed according to disease severity at that date.
Blinding	FISH analysis was conducted by independent expert pathologists prior to initiation of this study and were thus not aware of study goals. Stained sections were assessed by veterinary anatomic pathologists blinded to transgenic strain and/or treatment group. In RNA-sequencing studies, investigators were blinded to group allocation and study endpoints. Veterinary mouse technicians determining euthanasia criteria were blinded to transgenic status, treatment enrollment group, and disease status. For therapeutic studies, investigators determining group allocation were blinded to therapeutic received by each group.

Reporting for specific materials, systems and methods

We require information from authors about some types of materials, experimental systems and methods used in many studies. Here, indicate whether each material, system or method listed is relevant to your study. If you are not sure if a list item applies to your research, read the appropriate section before selecting a response.

Materials & experimental systems

n/a	Involved in the study
☐	☒ Antibodies
☒	☐ Eukaryotic cell lines
☒	☐ Palaeontology and archaeology
☐	☒ Animals and other organisms
☐	☒ Human research participants
☐	☒ Clinical data
☒	☐ Dual use research of concern

Methods

n/a	Involved in the study
☒	☐ ChIP-seq
☐	☒ Flow cytometry
☒	☐ MRI-based neuroimaging

Antibodies

Antibodies used	Fluorescent in-situ Hybridization (FISH) was performed with IGH/CCND1 XT, IGH break apart (Abbott Molecular, Downers Grove, IL) and MTCP1 (Empire Genomics, Williamsville, NY) probes.<br>Immunophenotyping of tumor cells in peripheral blood, spleen, and lymph node of Eμ-MTCP1 and Eμ-TCL1 mice by flow cytometry was performed as follows: APC rat anti-mouse CD45 (BD Biosciences Cat #559864), FITC rat anti-mouse CD45R/B220 (BD Biosciences Cat #553088), BV421 rat anti-mouse CD19 (BD Biosciences Cat #562701), PE rat anti-mouse CD5 (BD Biosciences Cat #553023).<br>Immunomodulatory assessment of Eμ-MTCP1 peripheral blood by flow cytometry was performed as follows: PE hamster anti-mouse CTLA4 (BD Biosciences Cat #553720), PE hamster IgG1 κ isotype control (BD Biosciences Cat #553972), BV421 rat anti-mouse CD5 (BD Biosciences Cat #562739), FITC rat anti-mouse CD45 (BD Biosciences Cat #553080), Alexa Fluor® 647 rat anti-mouse CD19 (BD Biosciences Cat #557684), LIVE/DEAD™ fixable near-IR dead cell stain (ThermoFisher Scientific Cat #L34976), rat anti-mouse CD16/CD32 (Mouse BD Fc Block™; BD Biosciences Cat #553142). BV510 hamster anti-mouse CD3e (BD Biosciences Cat #563024), BV650 rat anti-mouse CD11b (BD Biosciences Cat #653402), BB515 rat anti-mouse CD19 (BD Biosciences Cat #564509), BUV737 rat anti-mouse CD5 (BD Biosciences Cat #612809), APC rat anti-mouse CD93 (Biolegend Cat #136510), BUV395 rat anti-mouse CD45R/B220 (BD Biosciences Cat #563793), BV786 rat anti-mouse IgM (BD Biosciences Cat #564028), PerCP-Cy5.5 rat anti-mouse CD21 (Biolegend Cat #1234160), BV711 Rat anti-mouse CD23 (BD Biosciences Cat #563987), BV605 Rat anti-mouse IgD (BD Biosciences Cat #563003).<br>Histopathology staining was conducted with hematoxylin and eosin (H&E; Leica) F4/80, B220, and CD3 IHC.
Validation	FISH was done according to the manufacturer's recommendations, except prior to hybridization slides were pretreated with pepsin and postfix solution.<br>Gating strategies followed published data and technical resource publications [refs 59-61] and were adapted to allow exclusion and interrogation of CD19+CD5+ CLL-like populations. Fluorescence-minus-one (FMO) controls were used for each marker and gate position. Cells were gated on viable single mononuclear cells.<br>No customized antibodies were used.<br>All antibodies used for flow cytometry are from commercial sources (BD Biosciences, Biolegend, ThermoFisher Scientific). Validation data are available on the manufacturer's website and datasheets:<br><br>APC rat anti-mouse CD45 (BD Biosciences Cat #559864) → https://www.bdbiosciences.com/en-us/products/reagents/flow-cytometry-reagents/research-reagents/single-color-antibodies-ruo/apc-rat-anti-mouse-cd45.559864

FITC rat anti-mouse CD45R/B220 (BD Biosciences Cat #553088) → https://www.bdbiosciences.com/en-us/products/reagents/flow-cytometry-reagents/research-reagents/single-color-antibodies-ruo/fitc-rat-anti-mouse-cd45r-b220.553088

BV421 rat anti-mouse CD19 (BD Biosciences Cat #562701) → https://www.bdbiosciences.com/en-us/products/reagents/flow-cytometry-reagents/research-reagents/single-color-antibodies-ruo/bv421-rat-anti-mouse-cd19.562701

PE rat anti-mouse CD5 (BD Biosciences Cat #553023) → https://www.bdbiosciences.com/en-us/products/reagents/flow-cytometry-reagents/research-reagents/single-color-antibodies-ruo/pe-rat-anti-mouse-cd5.553023

PE hamster anti-mouse CTLA4 (BD Biosciences Cat #553720) → https://www.bdbiosciences.com/en-us/products/reagents/flow-cytometry-reagents/research-reagents/single-color-antibodies-ruo/pe-hamster-anti-mouse-cd152.553720

PE hamster IgG1 κ isotype control (BD Biosciences Cat #553972) → https://www.bdbiosciences.com/en-us/products/reagents/flow-cytometry-reagents/research-reagents/flow-cytometry-controls-and-lysates/pe-hamster-igg1-isotype-control.553972

BV421 rat anti-mouse CD5 (BD Biosciences Cat #562739) → https://www.bdbiosciences.com/en-us/products/reagents/flow-cytometry-reagents/research-reagents/single-color-antibodies-ruo/bv421-rat-anti-mouse-cd5.562739

FITC rat anti-mouse CD45 (BD Biosciences Cat #553080) → https://www.bdbiosciences.com/en-us/products/reagents/flow-cytometry-reagents/research-reagents/single-color-antibodies-ruo/fitc-rat-anti-mouse-cd45.553080

Alexa Fluor® 647 rat anti-mouse CD19 (BD Biosciences Cat #557684) → https://www.bdbiosciences.com/en-us/products/reagents/flow-cytometry-reagents/research-reagents/single-color-antibodies-ruo/alexa-fluor-647-rat-anti-mouse-cd19.557684

LIVE/DEAD™ fixable near-IR dead cell stain (ThermoFisher Scientific Cat #L34976) → https://www.thermofisher.com/order/catalog/product/L34976#/L34976

rat anti-mouse CD16/CD32 (Mouse BD Fc Block™; BD Biosciences Cat #553142) → https://www.bdbiosciences.com/en-us/products/reagents/flow-cytometry-reagents/research-reagents/single-color-antibodies-ruo/purified-rat-anti-mouse-cd16-cd32-mouse-bd-fc-block.553142

BV510 hamster anti-mouse CD3e (BD Biosciences Cat #563024) → https://www.bdbiosciences.com/en-us/products/reagents/flow-cytometry-reagents/research-reagents/single-color-antibodies-ruo/bv510-hamster-anti-mouse-cd3e.563024

BV650 rat anti-mouse CD11b (BD Biosciences Cat #563402) → https://www.bdbiosciences.com/en-us/products/reagents/flow-cytometry-reagents/research-reagents/single-color-antibodies-ruo/bv650-rat-anti-cd11b.563402

BB515 rat anti-mouse CD19 (BD Biosciences Cat #564509) → https://www.bdbiosciences.com/en-us/products/reagents/flow-cytometry-reagents/research-reagents/single-color-antibodies-ruo/bb515-rat-anti-mouse-cd19.564509

BUV737 rat anti-mouse CD5 (BD Biosciences Cat #612809) → https://www.bdbiosciences.com/en-nz/products/reagents/flow-cytometry-reagents/research-reagents/single-color-antibodies-ruo/buv737-rat-anti-mouse-cd5.612809

APC rat anti-mouse CD93 (Biolegend Cat #136510) → https://www.biolegend.com/en-us/search-results/apc-anti-mouse-cd93-aa4-1-early-b-lineage-antibody-6621?GroupID=BLG8671

BUV395 rat anti-mouse CD45R/B220 (BD Biosciences Cat #563793) → https://www.bdbiosciences.com/en-us/products/reagents/flow-cytometry-reagents/research-reagents/single-color-antibodies-ruo/buv395-rat-anti-mouse-cd45r-b220.563793

BV786 rat anti-mouse IgM (BD Biosciences Cat #564028) → https://www.bdbiosciences.com/en-us/products/reagents/flow-cytometry-reagents/research-reagents/single-color-antibodies-ruo/bv786-rat-anti-mouse-igm.564028

PerCP-Cy5.5 rat anti-mouse CD21 (Biolegend Cat #123416) → https://www.biolegend.com/en-us/products/percp-cyanine5-5-anti-mouse-cd21-cd35-cr2-cr1-antibody-5795?GroupID=BLG5432

BV711 Rat anti-mouse CD23 (BD Biosciences Cat #563987) → https://www.bdbiosciences.com/en-us/products/reagents/flow-cytometry-reagents/research-reagents/single-color-antibodies-ruo/bv711-rat-anti-mouse-cd23.563987

BV605 Rat anti-mouse IgD (1/100 dilution; BD Biosciences Cat #563003) → https://www.bdbiosciences.com/en-us/products/reagents/flow-cytometry-reagents/research-reagents/single-color-antibodies-ruo/bv605-rat-anti-mouse-igd.563003

Animals and other organisms

Policy information about studies involving animals; ARRIVE guidelines recommended for reporting animal research

Laboratory animals

Transgenic Eμ-MTCP1 mice were generated on a C57BL/6NTac background at The Ohio State University Comprehensive Cancer Center's Transgenic Mouse Facility. Mice were housed in an environment with 12-hour light/12-hour dark cycle and temperatures were maintained at 75°F +/- 2°F with 30-60% humidity. Food and water were accessible at all times. Mice were grouped into cages and provided enrichment whenever possible.

An equal ratio of male and female mice were maintained throughout all analyses. Adoptive transfer studies were conducted using 2 month old immune competent C57BL/6NTac mice.
Eμ-MTCP1 littermate mice were randomized and enrolled to receive continuous ibrutinib (~30 mg/kg/day via drinking water) or vehicle administration beginning at 2 months of age orally via supplemented drinking water. Mice were followed for leukemia onset and overall survival until reaching predefined removal criteria.

Wild animals	This study did not involve wild animals.
Field-collected samples	This study did not involve field-collected samples.
Ethics oversight	All experiments were carried out under protocols approved by The Ohio State University Institutional Animal Care and Use Committee (IACUC #2010A00000152-R3). Pre-defined euthanasia criteria for mice in all transgenic colonies and murine transplant models included lethargy, impaired motility, splenomegaly, enlarged lymph nodes, decrease in body weight (>20%), development of tumor masses, ruffled fur, hunched back, failure to nest, and loss of appetite.

Note that full information on the approval of the study protocol must also be provided in the manuscript.

Human research participants

Policy information about studies involving human research participants

Population characteristics	1744 suspected CLL specimens collected between November 2003 and December 2014 at The Ohio State University Comprehensive Cancer Center were evaluated for possible Xq28 rearrangements by screening metaphase karyotypes collected at time of initial biopsy. Covariate-relevant characteristics collected include: age at diagnosis (yrs), sex (M/F), diagnosis (CLL or other hematologic malignancy), IGHV mutation status (un-mutated/mutated), previous treatment (yes/no), and complexity (number aberrations).
Recruitment	Peripheral blood or bone marrow from patients with suspected CLL was obtained after written informed consent in accordance with the Declaration of Helsinki and under a protocol approved by the institutional review board (IRB) at The Ohio State University. Participants were not prospectively identified for this study. The population is representative for a tertiary referral center without any identifiable bias.
Ethics oversight	Protocol was approved by the Ohio State University

Note that full information on the approval of the study protocol must also be provided in the manuscript.

Clinical data

Policy information about clinical studies

All manuscripts should comply with the ICMJEguidelines for publication of clinical research and a completedCONSORT checklist must be included with all submissions.

Clinical trial registration	A retrospective analysis evaluating MTCP1 expression in CLL patients was conducted using two independent chemoimmunotherapy trial cohorts for which microarray data have been previously reported (CALGB '9712' - NCT00003248; CALGB '10101' - NCT00098670).
Study protocol	Full trial protocols for CALGB studies '9712' and '10101' can be found at Clinicaltrials.gov: NCT00003248 and NCT00098670. Blood samples for metaphase karyotyping were collected and screened under protocol IRB 2014C0126 at The Ohio State University.
Data collection	1744 suspected CLL specimens collected between November 2003 and December 2014 at The Ohio State University Comprehensive Cancer Center were evaluated for possible Xq28 rearrangements by screening metaphase karyotypes collected at time of initial biopsy. Translocations involving Xq28 were confirmed by two independent cytogeneticists.
Outcomes	Pre-defined outcomes and measurements for CALGB studies '9712' and '10101' can be found at Clinicaltrials.gov: NCT00003248 and NCT00098670. Primary and secondary outcomes were defined prior to study initiation and assessed by investigators according to National Cancer Institute Working Group (NCIWG) and National Cancer Institute (NCI) Common Toxicity Criteria (CTC) Version 2.0 guidelines.

Flow Cytometry

Plots

Confirm that:

- [x] The axis labels state the marker and fluorochrome used (e.g. CD4-FITC).
- [x] The axis scales are clearly visible. Include numbers along axes only for bottom left plot of group (a 'group' is an analysis of identical markers).
- [x] All plots are contour plots with outliers or pseudocolor plots.
- [x] A numerical value for number of cells or percentage (with statistics) is provided.

Methodology

Sample preparation	Peripheral blood from Eμ-MTCP1 and Eμ-TCL1 transgenic mice was collected monthly via check punch. Cells from the spleens

	of Eμ-MTCP1 and Eμ-TCL1 having met predefined euthanasia criteria were processed and isolated using methods previously described. Mouse B cells were isolated from whole spleen suspensions using EasySepTM mouse pan B cell isolation kit (STEMCELL Technologies; Cat #19844).
Instrument	Immunophenotyping was conducted using Beckman Coulter Gallios 3-laser-10-color (B5-R3-V2) cell analyzer and BD LSRFortessa Cell Analyzer (Cat #649225).
Software	All flow cytometry data were analyzed using KALUZA v2.0 software (Becton Dickinson).
Cell population abundance	Typically labeled 5x10^5 - 1x10^7 cells.
Gating strategy	Gating strategies followed published data and technical resource publications [ref 59-61] and were adapted to allow exclusion and interrogation of CD19+CD5+ CLL-like populations. Initial gating on whole mouse blood used FSC/SSC to include cell populations and exclude debris. Single cell populations were then gated using FSC/FSC to exclude irregular shaped cells or doublets. Single cells were then gated on count/CD45+ to include lymphocyte populations for further interrogation.

☒ Tick this box to confirm that a figure exemplifying the gating strategy is provided in the Supplementary Information.
